# The Sanitary Condition of the United States Volunteers

**Published:** 1862-10

**Authors:** 


### ART. III.—THE SANITARY CONDITION OF THE UNITED STATES VOLUNTEERS.

WHENEVER the history of the great civil war now raging in the United States shall be written, none of the many remarkable events which have distinguished its origin and progress will be more worthy of note by the historian; none will throw a more intimate light upon the constitution of that vast force with which the Northern States have maintained the struggle; none will so greatly redound to the honour of the Federal cause, as the formation and operations of that self-instituted, semi-official body, by which the provisions of the government for the welfare of the soldier and physical efficiency of the army have been supplemented—THE SANITARY COMMISSION.

The history and objects of this Commission, and the mode in which those objects have been attained, are of the highest interest to the student of military hygiéne. They are, moreover, of peculiar moment to us at the present moment, as from them we may learn the chief existing defects in the organization of our own volunteer forces, and the extraordinary importance of systematically seeing to the removal of those defects. They also teach us in what manner voluntary aid will probably have to supplement State efforts in maintaining the health and efficiency of our volunteers in the field, should it ever be their lot to be called upon for active service. The history and operations of the Commission, in short, teach us the necessity of systematic foresight if, under such circumstances, we would escape needless sacrifices of health and life—sacrifices more wide-spread, deadlier, and more disastrous than those of a foughten field. It is, indeed, chiefly from the light which the history of the Sanitary Commission throws upon the requirements of volunteer forces in the field, that we are induced to enter upon this subject with the comparatively imperfect materials still at our command. After no little difficulty and disappointment we have procured many of the papers published by the Commission, but these refer principally to its origin and earlier operations. Although, however, we could have wished, for the better completion of our purpose, to possess also authoritative accounts of its later operations, yet we should be loth not to make immediate use of such papers as we have already obtained; and it will be seen that from these we may readily deduce the most important particulars of that great lesson which we are anxious should be learned. If it be said that no just parallel can be drawn between the circumstances which led to the formation of our own and those which led to the formation of the United States volunteers, and the consequent condition and probable efficiency of the two bodies, we may say that no such parallel is sought to be drawn. But the principles which should control the organization of these and all like forces, and without which their complete efficiency is an impossibility, are essentially the same; and what these principles are may undoubtedly be best learned from carefully observing the unexampled experience of the Federal volunteers. These, lacking all those opportunities of careful and-long continued preparation possessed by our own volunteers, were hastily raised and organized, and almost immediately hurried into the field, and as a consequence all the peculiarities and evils of volunteer service were displayed in their most glaring characters, and on a gigantic scale. If it be assumed that these evils would be avoided by the better training which, happily, from our more fortunate position, our own volunteer forces have undergone and are still undergoing, we would simply remark that this is true only if the assumption includes systematic training in *all* that may be required of the volunteer in camp as well as at drill and on parade; in war as well as at peace. The tremendous catastrophe of the winter of 1854-55 in the Crimea, showed how utterly unavailing the highest degree of training and efficiency in arms was in staving off those disastrous evils which arose out of the neglect of systematic training in the requisite duties of a camp, and the domestic concerns of a soldier's life. But it is to these very evils that the volunteer will be most exposed in the field, but with which he is least able to contend, and to which he is much more liable to succumb than the regular soldier.

There is occasionally a disposition evinced to look upon the discipline of a volunteer as requiring much less of strictness than is adopted in the case of the professional soldier; to regard the volunteer as a sort of "irregular" soldier, to whom the stern control exercised over the "regular," would be most unfittingly applied. This may be correct, perhaps, in so far as it would apply to attempts to obtain from the volunteer that minute knowledge of military drill which we look for in the "regular;" but it is a vital error if it is advanced as an apology for any laxity in what the volunteer is justly called upon to learn. In this respect unfaltering exactitude is as requisite from him as the professional soldier, if he would hope to prove efficient in the field. Moreover, if he has become possessed with the notion that camp-life is a species of serious "pic-nic," the sooner he gets rid of the idea and addresses himself to a sober study of what camp-life means, the better for himself and his subsequent career under canvas. It is very pleasant, no doubt, to read of the apparently rollicking and hardy life led by several of the volunteer and irregular forces which have made themselves famous in history; but it must be borne in mind that in almost every instance where such forces have escaped grave disasters from privation and disease, the habits of life and character of food in camp have differed but very slightly from those which the volunteer was accustomed to in his own home. It is the entire change not only in habits, but in the character and preparation of the food to which our own volunteers would be subjected, if they were called into the field, which makes it of so great importance that in these respects they should be subjected to a systematic course of tuition, as well as in the use of their rifles. This all-important conclusion stands foremost in the lesson to be learned from the internal condition of the Federal volunteers and camps.

In May, 1861, a conjoint committee of three associations in New York, the "Women's Central Association of Relief for the Sick and Wounded of the Army," the "Advisory Committee of the Boards of Physicians and Surgeons of New York," and the "New York Medical Association for furnishing supplies in Aid of the Army," presented an address, in the name of those associations, to the Secretary for War, asking for "some positive recognition of their existence and efforts." The address stated that the associations were engaged at home in a common object, and were acting together with great efficiency and harmony to contribute towards the comfort and security of the troops by methodizing the spontaneous benevolence of the City and State of New York; obtaining information from the public authorities of the best method of aiding the War Department with such supplies as the regulations of the army did not provide, or the sudden and pressing necessities of the time did not permit the department to furnish; and in general, striving to play into the hands of the regular authorities in ways as efficient and as little embarrassing as extra-official co-operation could be. The associations asked then for some recognition for the purpose of facilitating their communications with, and as "essential to the peace and comfort of," the several bureaus of the War Department. "Would not," the committee urged, among other reasons, "the department win a still higher place in the confidence and affections of the good people of the loyal States and find itself generally strengthened in its efforts, by accepting in some positive manner the services of the associations we represent, which are labouring to bring into system and practical shape the general zeal and benevolent activity of the women of the land in behalf of the army? And would not a great economy of time, money, and effort be secured by fixing and regulating the relations of the volunteer associations to the War Department, and especially the Medical bureau?"

The committee asked, therefore, "that a mixed Commission of civilians, distinguished for their philanthropic experience and acquaintance with sanitary matters, of medical men, and of sanitary officers, be appointed by the Government, who shall be charged with the duty of investigating the best means of methodizing and reducing to practical service the already active but undirected benevolence of the people toward the army; who shall consider the general subject of the prevention of sickness and suffering among the troops, and suggest the wisest methods which the people at large can use to manifest their good-will towards the comfort, security, and health of the army."

It was further urged in support of this request, that commissions of a somewhat similar character had *followed* the Crimean and Indian wars; but that "the civilization and humanity of the age and of the American people demand that such a commission should *precede* our second War of Independence. We wish (continued the address) to prevent the evils that France and England could only investigate and deplore. This war ought to be waged in a spirit of the highest intelligence, humanity, and tenderness for the health, comfort, and safety of our brave troops. And every measure of the Government that shows its sense of this will be eminently popular, strengthen its hands, and redound to its glory at home and abroad."

In addition to the request for a commission, the Secretary at War was also urged to order a more vigorous inspection of volunteer troops; to adopt some measure for improving their cooking; to authorize the reception in the Army General Hospitals of the services of trained nurses; and to instruct the Medical bureau in case of need to call to the aid of the regular medical force, a set of volunteer dressers, drilled by the hospital physicians and surgeons of New York.

In support of the first of these requests, it was stated that under the State regulations then in force "a great number of under-aged and unsuitable persons are mustered, who are likely to swell the bills of mortality in the army to a fearful per-centage, to encumber the hospitals and embarrass the columns." An inspection of the troops, or a summary discharge of those obviously destined to succumb to the diseases of the approaching summer, was therefore asked for.

In support of the second request, equally important and significant arguments were brought forward. "The committee," the address states, "are convinced by the testimony of the Medical bureau itself, and the evidence of the most distinguished army officers, including the Commander-in-Chief, Adjutant-General Thomas, and the Acting Surgeon-General, that the cooking of the volunteer and new regiments in general is destined to be of the most crude and perilous description, and that no preventive measure could be so effectual in preserving health and keeping off disease, as an order of the department requiring a skilled cook to be enlisted in each company of the regiments. The Woman's Central Association, in connexion with the Medical Boards, are prepared to assume the duty of collecting, registering, and instructing a body of cooks, if the department will pass such an order, accompanying it with the allotment of such wages as are equitable."

Fully as practical were the reasons urged for the adoption of the third request. The Woman's Central Association of Relief, it was stated, had selected, and were selecting, out of several hundred candidates, one hundred women, suited in all respects to become nurses in the general hospitals of the army. These women the physicians and surgeons of the various hospitals in New York had undertaken to educate and drill in a most thorough and laborious manner; and the committee asked that the War Department should consent to receive, on wages, these nurses, in such numbers as the exigencies of the campaign required. It was proposed that the nurses should not advance to the seat of war until directly called for by the Medical bureau, or that the Government should be at any expense until they were actually in service.

The dressers suggested in the fourth request were to be young medical men, to whom such subsistence and recognition should be given as the rules of the service might permit under a generous construction.

The committee finally urged, that a Commission would bring these and others matters of great interest to the troops into the shape of easy and practical adoption; but if a Commission were not appointed, it was suggested that the Secretary for War should order the several suggestions made to be carried into immediate effect, if consistent with the laws of the department, or possible without the action of Congress.

This singularly interesting and prescient address of the conjoint Committee was the germ of the Sanitary Commission. It was signed by Henry W. Bellows, D.D., W. H. Van Buren, M.D., Elisha Harris, M.D., and J. Harsen, M.D., and it was dated the 18th May.

On the 22nd of that month a letter was addressed to the Secretary of War, by the Acting Surgeon-General, R. C. Wood, which recommended the appointment of a Commission, to be styled "A Commission of Inquiry and Advice in respect of the Sanitary Interests of the United States Forces." The letter pointed out that the Medical bureau was unequal to the great and urgent pressure which had fallen upon it, and that important and useful aid might be derived from the counsels and well-directed efforts of such a Commission. "This Commission," says the letter, "is not intended to interfere with, but to strengthen the present organization introducing and elaborating such improvements as the advanced stage of medical science might suggest; more particularly as regards the class of men who, in this war of sections, may be called to abandon the comforts of home, and be subject to the privations and casualties of war." The Acting Surgeon-General adopted the views expressed "in a crude and hasty manner" (as he states), in the address of the Committee of the three Associations, dated the 18th of the month; and he suggested that the Commission should be constituted of five gentlemen, whose names he gave, and who should have associated with them an officer of the Army Medical Staff.

On the 23rd of May the Committee of the three Associations presented to the Secretary of War a draft of the powers asked from the Government for the proposed Commission. This draft set forth that as the Commission would be organized solely for the purposes of inquiry and advice, no legal powers were sought, but only the official recognition and moral countenance of the Government which would be secured by the public appointment, as well as such a recommendatory order as would facilitate correspondence with the different officers of Government whom the Commission might be thrown into contact with. No pecuniary remuneration was asked for, but simply a room in one of the public buildings in which the Commission might meet, stationery, and "other necessary conveniences." It was also asked that the Commission should have leave to sit through the war, either in Washington or where its sittings might be deemed most useful; but it was promised that the Commission should disband if its operations proved embarrassing to the Government or less useful and necessary than was anticipated. The objects of the Commission were to supplement and aid in every practical way, without interfering with, the operations of the Medical bureau, and it proposed to effect these objects by instituting, first, an inquiry into the *materiel* of the volunteer force, "with reference to the laws and usages of the several States in the matter of inspection, with the hope of assimilating their regulations with those of the army proper, alike in the appointment of medical and other officers, and in the rigorous application of just rules and principles to recruiting and inspection laws." It proposed also to inquire "with scientific thoroughness into the subject of Diet, Cooking, Cooks, Clothing, Tents, Camping, Grounds, Transports, Transitory Depôts, with their exposures, Camp Police, with reference to settling the question—How far the regulations of the army proper are or can be practically carried out among the Volunteer

Regiments, and what changes or modifications are desirable from their peculiar character and circumstances? Everything appertaining to outfit, cleanliness, precautions against damp, cold, heat, malaria, infection; crude, unvaried or ill-cooked food, and an irregular or careless regimental commissariat, would fall under this head." Finally, the Commission proposed to inquire into the organization of Military Hospitals, general and regimental—"the precise regulations and routine through which the services of the patriotic women of the country may be made available as nurses; the nature and sufficiency of hospital supplies; the method of obtaining and regulating all other extra and unbought supplies contributing to the comfort of the sick; the question of ambulances and field service, and of extra medical aid; and whatever else related to the care, relief, or cure of the sick and wounded—the investigation being guided by the highest and latest medical and military experience, and carefully adapted to the nature and wants of our immediate army, and its peculiar origin and circumstances."

Such was the comprehensive scheme of the Commission, and on the 9th of June the proposal received the approval of the Secretary of War, and subsequently was confirmed by the President.

The Commission, more numerous than suggested by Acting Surgeon-General Wood, and under the presidency of the Rev. H. W. Bellows, D.D., forthwith drew up and submitted a detailed plan of organization to the Secretary of War, which plan received his approval. The Commission was to be divided into two committees, the one to be devoted to *Inquiry*, the other to *Advice*; and each committee was to be subdivided into three sub-committees. The sub-committees of Inquiry would devote themselves respectively to (1) "such obvious recommendations as an intelligent foresight and an ordinary acquaintance with received principles of sanitary science would enable the board at once to urge upon the public authorities;" (2) to the actual exploration of camps, hospitals, quarters, and transports, and to ascertaining the true condition and wants of the troops; (3) to an investigation of the domestic economy and intimate internal condition of the different forces. The duties of the sub-committees of Advice would be respectively, (1) to be the means of communication to the Central Government authorities of the counsels and suggestions of the Commission; (2) to look after the manner in which the orders, of a sanitary kind, of the War Department and Medical bureau were carried out, and secure as far as practicable by explaining and enforcing upon ignorant, inexperienced, and careless officials the actual accomplishment of the regulations issued; (3) to maintain direct relations with the State governments and with the public associations of benevolence; to secure uniformity of plans, and their proportion and harmony of action; and finally, abundance of supplies in moneys and goods, for such extra purposes as the laws do not and cannot provide for—to organize, in fact, methodize, and reduce to serviceableness "the vague, disproportioned, and haphazard benevolence of the public."

This briefly was the plan of working adopted and immediately carried into effect by the Commission. Funds were at once sought through the medium of voluntary subscriptions, and the early circulars of the Commission, asking for pecuniary aid, are not a little characteristic of the foresight, energy, and thorough practicality of purpose with which it entered upon its self-imposed and noble work. The first circular was addressed to the presidents and officers of the various Life Insurance Companies of the United States. In this circular it is said:—

"We look, then, to the Life Insurance Companies, whose intelligent acquaintance with vital statistics constitutes them the proper and the readiest judges of the necessity of such a Commission, to give the first endorsement to our enterprise by generous donations—the best proof they can afford the public of the solid claim we have on the liberality of the rich, the patriotic, and the humane. We beg to remind you, moreover, that even those Life Insurance Companies which have no war risks outstanding are directly and deeply interested in promoting the objects of this Commission. For no fact in medical history is better established than this—that diseases breaking out among soldiers in camp or garrison, for the want of prudent sanitary measures, and extending among them on any considerable scale, are soon shared by the community at large. The mere presence in any country of an army extensively infected is a centre of poison to its whole people. If pestilence do not break out (as it commonly does) ordinary maladies assume a malignant and unmanageable type, and the general ratio of mortality is heightened in a fearful degree."

In a circular addressed to the public generally, the Commission say:—

"It is supposed that fifty thousand dollars could be expended with the greatest advantage during the present year in the work of the Commission, and that every single dollar so spent would save one life. Every dollar less than this placed at the disposal of the Commission must be supposed a needless exposure and probable loss of life . . . . It is hardly necessary to suggest that every soldier who survives the exposure of the next four months will be worth, for military purposes, two fresh recruits; that every man lost by neglect makes a complaining family, and forms a ground of unpopularity for the war; that every sick man deprives the ranks of one or two well men detailed to take care of him; that pestilence will demoralize and frighten those whom armed enemies cannot scare; that the men now in the field are the flower of the nation; that their places cannot be filled, either at home or in the ranks; and that the economical, the humane, the patriotic, the successful conduct of the war, and its speedy termination, is now more dependent on the *health* of the troops than any and all other conditions combined."

This was written in June, 1861, and how terribly its truth has been illustrated by the events of the Federal campaign before Richmond in the present summer, must be apparent even to those who have noted least carefully the news from General M'Clellan's army.

While energetically seeking pecuniary help from the public, the Commission also increased the magnitude of its operations by electing and obtaining the co-operation of associated members in the different States of the Union; and at the same time the President and Secretary of the Commission and Dr. Harris personally inspected many of the volunteer camps. The result of this preliminary inspection fully confirmed the justness of the reasons which had led to the formation of the Commission, and the great necessity which existed for its formation.

The President reported in the highest terms of the physical and moral qualifications of the men constituting the forces in the West—in Camp Dennison, near Cincinnati, Camp M'Clellan, at Cairo, and Camp Pope, at Alton—and of the self-devotion of the officers; but he adds:—

"They have the best of purposes; but they know not yet how to control the diet, the personal habits, the ventilation and police of their quarters and camps. They are studying war *tactics*, intent on making *soldiers;* they rashly assume that intelligent men know how to take care of themselves; and they are already finding dysentery seizing their regiments with a most threatening grasp. The most striking difference is already apparent in camps and troops, according as attention is given or denied to the character of the water used, the situation of the camp with reference to the prevailing winds, and to the regulation of sinks, and the cleansing of tents and quarters. Two regiments, separated by a quarter of a mile only, contained, in one camp, not a dozen of sick men; in the other, two hundred and fifty men more or less ill with dysenteric diarrhœa, and all because one was on a plain with decent well-water at hand, the other in a wood, with a wretched puddle of black ditch-water as the only resource for drinking and cooking! Do you ask [he adds, and this is but one of many illustrations showing that circumlocution and red tape held as firm and evil a grasp over our cousins at the beginning of the war, as it ever did over ourselves]—do you ask, will medical men and officers, too, stand with folded arms and see this go on without immediate and energetic remonstrance and action? They will, I reply, under some provision of military etiquette, or some governmental obstacle, which it requires the boldness and decision of a power in confidential relation with the War Department to put aside."

The Secretary reported most unfavourably of the internal condition of many of the camps which came under his observation, and of the personal cleanliness of the soldiers. There was " often hardly a pretence," he states, " of performing the ordinary police duties of a military camp." The dress of the majority of the men is described as " inappropriate, unbecoming, uncomfortable, and not easily kept in a condition consonant with health. It is generally much inferior, in every desirable respect, to the clothing of the regulars, while it has cost more than theirs. . . . . The common kepi of the volunteers is pert, unsubstantial, ungraceful, uncomfortable, and dangerous." Of the food, he says: " It is doubtful if any army of the same size ever fared as well as to substantial articles of food for months together;" but he adds: " The raw materials furnished are generally atrociously cooked and wickedly wasted." The troops, however, were found to be suffering from want of vegetables. Diarrhœa was prevalent among them, attributed to this cause, and a case of scurvy had even then been reported near Washington, and several cases had been reported in the West. " The volunteer army is generally believed," is the significant comment, " to be in great danger of decimation by scurvy and dysentery." The deficiency in vegetables arose from the almost insuperable difficulties of obtaining the necessary transport, experienced even in the immediate vicinity of Washington. Of camp cooking, it is said, that " in no respect are the volunteers in so much need of instruction, advice, orders, and assistance, as in this." And it is suggested that, " perhaps the best way of meeting the difficulty would be at once to endeavour to obtain the services of sea-cooks from shipping ports, and attach them, one to a company, throughout the army." Finally, the general result of the Secretary's survey is summed up in the following important remarks, now of historical interest:—

" The Secretary must say, in conclusion, that he is compelled to believe that it is now hardly possible to place the volunteer army in a good defensive condition against the pestilential influences by which it must soon be surrounded. No general orders calculated to strengthen and guard against their approach can be immediately enforced with the necessary rigour. The captains especially have in general not the faintest comprehension of their proper responsibility; and if they could be made to understand they could not be made to perform the part which properly belongs to them in any purely military effort to this end. To somewhat mitigate the result is all that the Commission can hope to do."

As a practical pendant to this gloomy summary the Secretary suggests that certain limited administrative powers should be given to the Commission, in addition to their advising function, because if, without interfering with military discipline, the Commission and its agents possessed a claim upon the commander of a camp for the means at his disposal for abating the nuisance within it, much more could be done than otherwise would be the case. "At least," he adds, "there should be the right to require, where the advice of the Commission is disregarded for military reasons, that those reasons should be given in writing by the commanding officer to his military superior." Further, the Secretary, after remarking upon the willingness of officers in command to facilitate their inquiries, makes the following observations on the influence of camp inspectors and inspections, in inducing improvements in the sanitary condition of the forces:—

"The Secretary is at present of opinion that more is to be effected in the way of prevention by this agency than by any other means at the immediate command of the Commission. The business of such inspectors, if many should be employed, will need to be carefully systematized; they must be thoroughly instructed, and should be provided with printed advice upon various subjects of camp life and military duty, to be furnished as occasion may offer to officers of different grades, to cooks, and to privates. Thus presenting themselves to make official inquiry only, they will, without special effort or intention, really be the best possible missionaries of Sanitary science to the army. If there should be 300,000 men in the field—and it is thought that each regiment should be visited at least once a week on an average—twenty men of special qualifications for the duty would probably be needed as travelling inspectors."

On the 13th June, 1861, six of such inspectors were employed at various points from the Chesapeake to the Missouri, and their duties are thus stated in a paper published by the Commission at the date named:—

"These inspectors act under detailed instructions, and make weekly reports to the resident secretary of the Commission, (Frederick L. Olmsted, Esq.,) at Washington. Their duties are, generally, to visit the camps, barracks, quarters, and regimental hospitals, systematically and regularly, with a view to discover and remedy defects in their drainage, ventilation, &c., in the quality of the food and water supplied the men, in the system (if any) of camp cooking, in clothing, camp police, medicines, bedding, and hospital stores, in the supply of disinfectants, and in every other particular by which the health of the troops can be affected. It is the natural and excusable ignorance of a very large proportion of our newly-appointed volunteer officers on these and other sanitary points, and the fact that they do not appreciate the immense importance of attending to them daily and systematically, which constitute the chief source of peril to our troops. Officers generally take it for granted that their duty toward their men begins and ends with drill and parade, forgetting that camp disease is by far the most dangerous enemy they have to fear, and at the time the only enemy against which vigilance and precaution are almost certain of success. It is believed that the constant attention and care of intelligent and educated inspectors charged with the sole duty of watching over the sanitary condition of camps, &c., and of calling the attention of officers and men to the serious defects that are almost invariably found there, is the only available remedy for this evil.*

The actual and necessary expenses of these inspectors (travelling included) is estimated at not less than 1500 dollars per annum; and a salary was suggested of 600 dollars per annum over and above the necessary expenses. Up to the period referred to, however, the inspectors had served without any compensation beyond their actual expenses.

A resident inspector of general hospitals had also been appointed, and others were needed. His duty was to exercise supervision over the care of the volunteers in hospitals, and regulate the distribution of those extra comforts and requirements which were not to be obtained from the Government stores, but which were provided by the public bounty, and for which still increased funds were urgently asked for by the Commission. In the paper to which we have last referred, the Commission point out that although the hospital stores furnished by Government were excellent of their kind, the list of articles provided had been necessarily made up with reference solely to the wants of the regular army. "But," it is justly added, "among the rank and file of our volunteers are to be found representatives from every class in society, including many to whom certain additional comforts are matter of necessity; the want of such retards convalescence, if it does not prevent recovery, and those comforts the Commission hopes to be enabled in some degree to provide, without distinction, to all who need them."

The conclusions at which the Commission arrived from the details laid before them concerning the condition of the volunteer forces and camps, ascertained in this first inspection; and the recommendations, arising out of these conclusions, which they submitted to the Government, were embodied in a series of resolutions passed in the second, third, and fourth sessions of the Commission.

The resolutions and accompanying recommendations, had reference to many of the most essential points connected with the internal economy of the volunteer forces in the field. They included suggestions for a better method of paying the soldiers, improving the rations, providing cooks, the restriction of leave, the regulation of the sale of intoxicating drinks, the employment of nurses, the establishment and organization of

* "Before entering any camp they (the Inspectors) are required to obtain the formal approval of the Major-General, the Brigadier-General, and the Medical Director, in whose military jurisdiction it is included, together with an introduction to the commanding officer of the regiment, and through him to the company officers."

hospitals, the improvement of the *morale* of the soldiers, facilitating the communication of the wounded with their friends, and obtaining for them religious and entertaining books, the washing of the clothing of the troops, the sanitary government of camps, and so forth.

Several of the resolutions are deserving of special remark. In the resolution recommending that a better mode of paying the soldiers should be adopted, the secretaries of the Commission are instructed to "use all diligence in showing the moral bearing of such a measure on the health of the troops, and the comfort and self-respect of their households." In a subsequent resolution upon the same subject, its importance is again urged, from the consideration of "the mischief done to the physical health of the soldiers of volunteer regiments, by improvident expenditure of their pay; the wants of those dependent on them, who are in danger of becoming a public burden, and creating a vast pauper element in our cities and elsewhere; the danger of the war becoming unpopular in consequence; and the prospect of systematic private speculations in the soldiers' pay." The admirable foresight and philosophical estimation of the moral as well as physical dangers, and the remote as well as immediate relations of the soldier, so conspicuously manifested in these resolutions, are equally found in others.

It is also recommended that provision should be made by law for the enlistment or selection, in each company, of a cook, in addition to the detail from the ranks as then provided for; and that the said cook should receive the pay of a musician, and one ration per day, and be a non-combatant. It would be difficult to exaggerate the importance of such a provision in the case of all volunteers.

Another recommendation worthy of note is the addition of *butter*, whenever practicable, to the rations.

The importance also of a correct registration and frequent returns of deaths and interments is also insisted upon; and, finally (that is in our references, but not in the order of the resolutions), the following pregnant resolutions were adopted, which cannot be too strongly commended to the attention of all volunteer officers. They embody the primary secret of the physical and moral welfare of soldiers :—

"*Resolved*, That the Sanitary Commission, in their endeavours to promote temperance, cleanliness, and comfort among the troops, have become convinced that the first sanitary law in camp and among soldiers is *military* discipline; and that unless this is vigorously asserted and enforced, it is useless to attempt and impossible to effect, by any secondary means, the great end they propose—which is the health and happiness of the army.

"*Resolved*, That looking only to the health and comfort of the troops it is our profound conviction that any special relaxation of military discipline in favour of volunteer troops, based either upon their supposed unwillingness or inability to endure it, or upon the alleged expectation of the public, is a fallacious policy, and fraught with peril to the lives of the men and the success of the national cause; and that, speaking in the name of the families and the communities from which the volunteers come, and in the name of humanity and religion, we implore that the most thorough system of military discipline be carried out with the officers and men of the volunteer force, as the first and essential condition of their health, comfort, and morality.

"*Resolved*, That the health and comfort and efficiency of the men is mainly dependent on the uninterrupted presence, the personal watchfulness, and the rigid authority of the regimental and company officers; and that all the great defects, whether in the commissariat or in the police of camps, are radically due to the absence of officers from their posts and to the laxity of the discipline to which they are themselves accustomed—a laxity which would never be tolerated among regulars, and which, while tolerated among our soldiers, will make our force a crowd of armed men rather than an army.

"*Resolved*, That it is the public conviction of this Commission, that the soldiers themselves, *in their painful experience of the want of leaders and protectors*, would heartily welcome a rigid discipline exerted over their officers and themselves; that the public would hail with joy the inauguration of a decisive, prompt, and rigid rule, extending alike to officers and men; and that any despondency or doubt connected with our military and national prospects, or with the health and security of our troops, would disappear with the first indications of rigid order enforced with impartial authority throughout the whole army."

Let us turn now briefly to the reports we possess of the actual operations of the Commission. The latest of these is dated the 9th of January of the present year.

Five months after its formation the Commission had at work fourteen inspectors, each having a defined portion of the army under his observation, and six other gentlemen engaged in special duties, all men of scientific education, generally physicians of professional position and large experience, and its expenses averaged 5000 dollars a month. It had established a "Soldiers' Home" in Washington for the temporary reception of the sick and invalided, and for detached bodies of men, *in transitu*, and who had previously, in several instances, been exposed to great suffering from exposure occasioned by the want of such accommodation; its representatives had been well received by officers of every grade and by the soldiers; it had filled up several serious blanks in the provision for wounded and sick which had arisen from the inaptitude of the Medical bureau, hampered by its regulations, adapted only to the small force of the regular army; it had become the recognised agency of the bulk of that private bounty which was lavished by the different States upon the forces; and it had effected many ameliorations in the sanitary condition of the volunteers. There could be little doubt also that through its labours systematic attention to sanitary laws was becoming more generally understood to be a part of the duty of a military officer; and it was remarked that at this time the more recently enlisted regiments began the campaign better than those enlisted at the opening of the war, and improved faster. This improvement was to be attributed not only to the personal influence and instructions of the Inspectors, but in part to the divers publications of the Commission, calling the attention of officers and men to the precautions against disease, and which, to the numbers of more than one hundred and fifty thousand, had been scattered through the army and the country, and largely reprinted in the newspapers.

The numerous papers published by the Commission, in addition to its reports, are of great interest. Several of these documents now lie before us. One (No. 8) contains a series of queries on the constitution, internal condition, and requirements of the forces furnished by or encamped in the several States; a second (No. 9) is a carefully drawn up series of questions on the general hygienic condition, food, clothing, and camp police, of the different volunteer forces; and a third (No. 19 A) is an elaborate and most instructive form of camp inspection return. Another paper (No. $17^2$), also printed as a small 16mo. pamphlet, contains a well drawn up series of pithy "Rules for Preserving the Health of the Soldier," adapted for both officers and men. In addition to these papers there is a valuable series prepared for the use of the Volunteer Medical Staff. Assuming that the majority of the members of this staff would be called upon to do duties entirely novel to them, and under circumstances which would debar them from obtaining such special information as they might require for their guidance, one of the first tasks undertaken by the Commission was to have drawn up, by those best qualified for the purpose, detailed instructions concerning the peculiar duties which would be required from the volunteer surgeon, and the graver diseases and injuries with which he might have to contend, and respecting which his ordinary text-books would to a great extent fail him.

Among the papers of this series now before us, we have (A)* "A Report on Military Hygiene and Therapeutics," prepared by a Committee of the Surgical Section of the New York Academy of Medicine; (B) Directions to Army Surgeons on the Field of Battle, from the writings of Mr. Guthrie; and Reports of Committees on (E) Vaccination in Armies; (F) Amputations; (K)

* The capital letters are the distinguishing letters of the different papers referred to.

Continued Fevers; (M) Dysentery; (O) Fractures in Military Surgery; (P) the Nature and Treatment of Miasmatic Fevers; and (No. 31) on Quinine as a prophylactic against malarious diseases. There is a paper (No. 28), also headed "Advice as to Camping by the British Government Sanitary Commission," a reprint of the conclusions of Drs. Milroy and Sutherland and Mr. Rawlinson on this subject, in their report on the Sanitary Condition of the Army in the Crimea; also a paper by Dr. Valentine Mott, "the veteran chief of American Surgery," entitled "Pain and Anæsthetics," and advocating the more extended use of anæsthetics in naval and military practice.

The condition of the Volunteer Army in the months of September and October, 1861, is set forth in a report of the Commission, based upon returns from two hundred regiments. These, it is stated, accurately indicated the condition of the army in the particulars specified, "so far as the condition of the regiments in question, taken at random, and some from each division of the army in the field, was at the time of inspection fairly representative of the condition of the whole."

The time occupied in recruiting each of these regiments averaged six weeks, the shortest period being ten days, the longest about three months. Two-thirds of the volunteers were probably American born, and nine-tenths citizens, educated under the laws of the Union, and in the English tongue. In 76½ per cent. of the regiments inspected, native Americans constituted the majority; in 6½ per cent. there was a majority of Germans; in 5½ of Irish; and in 5½, also, the number of native born and foreign born was about equal: in 1 per cent. the returns gave no information on this point. The average age of the volunteers was estimated to be a little below 25 years; of the officers 34. In 58 per cent. of the regiments there had been no pretence of a thorough inspection of recruits on enlistment; and in only 9 per cent. had there been a thorough re-inspection when or after they were mustered Of 1620 men discharged from the army of the Potomac, as unfit for service, in October, 53 per cent. were discharged on account of disabilities that existed at the time and before their enlistment. Sufficient care had also not been taken to exclude from enlistment men "notoriously vicious and degraded." The Report rightly states that—

"In the regular service, persons of this class are, from the very moment of enlistment, controlled in some degree by the habits of command that have been acquired by their officers, and by the systematic and exact discipline they are thus enabled to enforce. But, among newly organized volunteers this cannot be expected, until the whole command has been for some considerable time in service, and until the majority of the men have become soldiers in reality, as well as in name. While the educational process is going on, the mere presence in camp of half a dozen dissolute, insubordinate, and ruffianly men tends very much to retard the progress in discipline of the whole command. They set an example of unwholesome indulgence of every kind, thwart all measures for the sanitary improvement of the camp, are the first subjects of disease, and the first to turn their backs on the enemy. Whatever disloyalty and desertion have occurred among our soldiers, may generally be traced to persons of this class. It is to be hoped that all such will hereafter be rigorously excluded from the people's army."

The Report also points out the importance of sanitary regulations being strictly enforced at the various depôts for volunteer troops; of every recruit being vaccinated immediately on enlistment; and of increased attention to the hygienic care of soldiers in transit by railroad and transports.

Camp sites had been generally selected for military reasons alone, and with little reference to sanitary considerations, the regimental surgeon being seldom consulted on the subject. In many instances disease was distinctly traceable to this omission. The artificial drainage of the camps was very imperfect, when first visited by the Inspectors, "the men of each tent being left in most cases to form drains around it according to their own judgment. As a consequence, the drains so constructed not unfrequently aggravated the evils they were designed to remedy." The camp arrangement was usually governed by the *Army Regulations*, but the tents were more closely placed together than the minimum there prescribed. The difficulty of efficient drainage and due cleanliness of the camp was thus increased. Six men were placed in a "wedge tent," and from twelve to sixteen, and sometimes twenty in the Sibley tent. The tents were seldom tolerably ventilated at nights. The difficulty of ventilating the "wedge" tents is considerable, and the Commission early warned the War Department of the evils which might arise from this source. The Sibley cannot be so tightly shut up as the wedge form. It was found by the inspectors that typhus was occurring more frequently in the latter than the former tents—the ratio being 29·5 in the "wedge," to 23 in the Sibley. In one volunteer regiment only, the 7th Massachusetts, under the medical charge of Surgeon Holman, had a thorough ventilation of the wedge tent been generally established. "It was here induced by the occurrence of typhoid fever, and by this, prominently among other means employed for the same end, the most gratifying and, at this season, unusual result of banishing this formidable disease has been obtained." The inspectors advised the striking of each tent once a week, for the purpose of giving it a perfect cleansing and airing, and the practice had been largely adopted.

Fifty-eight per cent. of the regiments had been provided with the wedge tent; 10, with the wall tent; 7, with the bell tent; and 19 with the Sibley; others not stated. Ninety per cent. of the tents were made of good canvas, the remainder of twilled cotton or drilling, or so old as to be leaky. Twenty-four per cent. of the regiments were provided with tent flooring of boards; 20 per cent. with india-rubber blankets; in 21 per cent. straw was used or branches, as a bed; and in 35 per cent. the men slept on the ground. The following table shows the ratio of sick men per thousand in regiments which had been supplied respectively with india-rubber blankets, wooden tent-floors, straw, fir boughs, or cedar boughs, and in those which had been sleeping on the ground. These data were taken from the returns of 120 regiments, and chiefly in November.

The forces in Western Virginia were, as a rule, unprovided with rubber blankets, and they suffered special hardships in other respects; they are, therefore, excluded from the comparison in the second column of the table. Moreover, as, up to the period when the data were collected, rubber blankets had not been issued by the Government, but provided solely by private bounty, it is not improbable that the regiments furnished with them were also better provided in other respects. It is also probable that those sleeping on the bare ground were generally at a greater distance from the supply depôts than the others, and consequently not as well provided for in several respects.

| Regiments Sleeping on | Entire Number of Regiments. | Those in Western Virginia excluded. |
|---|---|---|
| | Average Ratio of Sick per 1000. | Average Ratio of Sick per 1000. |
| Wood | 75·7 | 61·5 |
| India-rubber | 60·9 | 60·9 |
| Bare ground | 91·3 | 69·3 |
| Straw or fir-boughs | 77·5 | 45 8 |

A limited examination of the diseases of the army further showed, that the largest proportion of diseases of the typhoid type occurred with regiments sleeping on rubber blankets, the least with those on straw or boughs; the largest proportion of catarrhal affections, with regiments on wooden floors, the least with those on the ground; the largest of rheumatism, with those on wood, the smallest with those on straw or boughs; the largest of malarial, with those on the ground, the least with those on straw or boughs.

From these facts the Commission concluded, as they had before assumed, that the best bed for soldiers in camp was one formed from fir or cedar spray. The inspectors from the outset had been instructed to recommend its use when practicable. It was advised that the material should be frequently removed and burned, after a thorough cleansing of the tent floor, the tents being struck for the purpose.

The fact, however, of chief and more general interest to be deduced from these particulars is, that any of the three different forms of flooring is better for the soldier, so far as the whole amount of sickness is concerned, than the bare ground ; and the next best form to straw or fir boughs, is that which of all the others is most practicable, convenient, and fitted for general use, the india-rubber blanket.

The experienced officers of the volunteer forces objected to the board floors in tents. They regarded them as being damper than the ground itself, as affording opportunity for the collection of rubbish and dirt, and impeding the thorough cleansing of the tent site.

In 20 per cent. of the camps only were the latrines badly regulated ; in 23 per cent. the offal was imperfectly disposed of ; but in 50 per cent. the litter and manure of the horses had been allowed to accumulate an indefinite period, the stables being sometimes found actually within the camps.

Five per cent. of the camps inspected were in admirable order, and 45 per cent. were fairly clean and well policed ; but the condition of 26 per cent. was negligent and slovenly, and that of 24 per cent. decidedly bad, filthy, and dangerous.

In nothing were the volunteer officers more remiss in duty than in not ascertaining deficiencies of clothing among the men. Where deficiencies had not been made good, it was in "nearly every case owing to the ignorance, negligence, or knavery of regimental and company officers." There were ample supplies of all necessary articles in the principal depôts, from which all existing wants could be supplied at a short notice. "Never, probably," says the report, "was so large an army as well supplied at a similiar period of a great war."

Where regiments existed in which the men had but one shirt each or were badly provided with trousers (volunteers having been frequently seen during the summer on duty and on parade in their drawers only), the men were probably also in fault to no small extent. They often, in fact, sold or bartered away articles of dress issued to them.

The attention to cleanliness of person (even of the exposed parts) and clothing, was by no means what it should have been. "The volunteer army is more unsoldierlike in respect to matters of this kind, and its improvement has been slower in them than in any other." "Slovenliness," says the Report, "is our most characteristic national vice." Among the volunteers it was rare to find men provided with the few articles which were essential to an economical care of the clothing; still more unfrequently were they found possessed of those things which were requisite to the maintenance of health in crowded camps and quarters. "This want, also," says the report, "stands directly in the way of the development of that *esprit de corps* which is as essential to military efficiency as to health, and is the only reason which can be assigned for the greater difficulty which the inspectors have found in inducing a marked improvement in this direction than in any other." The Commission insists upon the sanitary importance of that minute attention to dress and equipment required from the regular forces, and upon the essential need of every man being provided with the necessaries of personal cleanliness, and the means of repairing his clothes and keeping it in good order. Even after typhus had entered camps, the Commission found it difficult to make the volunteer officers realize the actual military necessity upon which the army regulations, with reference to the personal cleanliness of the men, are based. "If," it is added, "five hundred thousand of our young men could be made to acquire something of the characteristic habits of soldiers in respect to the care of their habitations, their persons, and their clothing, by the training of the war, the good which they would afterwards do as unconscious missionaries of a healthful reform throughout the country, would by no means be valueless to the nation."

We have already mentioned the abundance of food, except vegetables, and the defective cooking, but in the latter respect great and constant improvement was observable. The arrangements for company and hospital funds were most imperfect, and the system of sutlers required to be much more stringently regulated. "In our regiment," reported one surgeon, "we have the best sutlers on the Potomac; *nevertheless they prove, in actual practice, an unmitigated curse.*" The Commission believed that the volunteers were more temperate than any European army. In the majority of the regiments there was very little dram drinking, except shortly after pay-day.

☞ In this Report, the resolutions of the Commission on the primary importance, as a sanitary measure, of enforcing military discipline in camp are reiterated. It is asserted, also, contrary to the opinion expressed by professional soldiers, that "it may fairly be concluded that a special military education is not at all necessary to adequate appreciation of the value of discipline or to the enforcement of discipline. . . ."

"There is, indeed, room for doubt [it is added] if the conviction which prevails with regular officers of the difficulty of enforcing discipline with volunteers, and their consequent hesitation and endeavour to accommodate their habits to the supposed necessity for moderation in the exercise of authority in dealing with volunteers, is not a greater hindrance to their progress in discipline than the inexperience of the officers chosen from among civilians." The report further says :—"Commiseration for what are erroneously considered technical offenders, and moderation or neglect in dealing with them, is costing the country more lives by far than the bullets of the enemy, and is adding many millions to the expense of the war."

There were great deficiencies in the provisions for the recreation of the men, and the officers had very imperfectly learned the importance of physical exercises in improving the morale of the forces; but very praiseworthy attempts had been made to form regimental bands. There was, moreover, an intense demand for books and periodicals in the different camps. In one case, sixty dollars had been subscribed by a company, from its ration savings, for newspapers, and a tent had also been obtained and set apart as a reading-room. About one-fifth of the regiments possessed libraries, mostly of religious books. They were generally the gift of the chaplain. Religious organizations existed in about half the regiments and were rapidly increasing. The American Tract Society alone had distributed among the soldiers more than 20,000,000 pages (equal to 60,000 12mo. volumes). "The number of letters written by the volunteers is remarkable, and a delightful indication of a fact which should remove all fear of a permanent military despotism in the country. In some regiments of 1000 men, it has averaged, for weeks, above 600 a-day. For all the regiments it must have been, through the summer, not far from 300." We have already referred to the deficiencies in the arrangements for the remittance of pay.

Of the medical staff it is reported that the surgeons of 176, out of the 200 regiments in question, were sufficiently well qualified; 13 of doubtful competence; 4 incompetent; and of 7 regiments no account was given. Moreover, 129 of the regimental surgeons were reported not only as competent, but as having discharged their duties with creditable energy and earnestness. The arrangement, equipment, and supplies of the regimental hospitals were good in 105 instances; indifferent or tolerable in 52; and bad in 26. In 13 regiments no hospitals had been organized.

The following table shows the aggregate strength of the two hundred regiments under consideration; the numbers sick in hospitals and in quarters ; and the proportion of such sick to every 1000 strength, and to every 1000 cases on the sick list :—

| Of 200 Regiments last visited, previous to November, 1861. | Aggregate Numbers. | Present Strength on Sick List. | |
|---|---|---|---|
| | | Proportion to every 1000 Strength. | Proportion to every 1000 Sick. |
| Strength when mustered ............... | 176·639 | | |
| Strength when inspected ............... | 176·042 | | |
| On sick list at the time of inspection | 12·841 | 73 | 1·000 |
| Sick in general hospital ............... | 2·756 | 16 | 215 |
| Sick in regimental hospital ............ | 2·973 | 17 | 231 |
| Sick in quarters ........................... | 7·112 | 40 | 554 |

In the army of the Potomac the average constant number of sick, per 1000, had been 63 ; in the department of Western Virginia, 162 ; and in the valley of the Mississippi, 116. The average constant number of sick during the months of August, September, and October, in the regiments east and west, so far as inspected, had been 77 per thousand. In this number all were included relieved from duty for any sort of physical indisposition however slight. At this rate, in order to secure a constantly active force of 300,000 men, the nation would have to maintain in the field an army of 325,000. The number of sick varied in different regiments from 0·33 per cent. to 49·0 per cent. The average length of time lost from active duty in each case reported was a little more than five days. The average health of the whole volunteer force was inferior to that of the regulars; but that of the army of the Potomac was somewhat better.

The following items of approximative comparison are given in the Report. In the whole British army, in time of peace, 6·5 per c . of the force otherwise available are constantly in hospital. Of the British army in the Peninsula, from 1808-1814, 21 per cent. were constantly "sick in hospital." The number of sick ranged from 9 to 33 per cent. of the whole force at different periods. In the British army in the Crimea, to maintain 100 effective soldiers in the field it was necessary to provide for 26 6 sick men. The annual rate of mortality was 3 per cent. from wounds, and 20 per cent. from disease.

The average mortality of the army of the Potomac had been during the summer at the rate of 3½ per cent. (allowance being made for those who die after their discharge from causes connected with army life). The mortality in the West was probably not far from 5 per cent.

The mortality from *disease* in the Royal navy of Great Britain is 140 per cent. greater in time of war than in peace, rising from an annual rate of 15 or 16 to one of 37 or 38 per 1000 strength.

The following statement exhibits a classification of the cases of disease in the volunteer army during a portion of the campaign, showing also the per centage of casualties of all kinds (wounds, accidents, &c.) for the same period, compared with like returns for the army of the Crimea, from April 10, 1854, to June 30, 1856:—

| | Army of Potomac. | Army of West. | Army of Crimea, April 10, 1854, to June 30, 1856. |
|---|---|---|---|
| Zymotic disease (per cent.) | 61·1 | 76·4 | 69·8 |
| Constitutional | 1·2 | 0·6 | 0·5 |
| Local | 30·7 | 17·3 | 15·6 |
| Developmental | 3·4 | 3·5 | 0·1 |
| Violence | 3·6 | 2·2 | 14·0 |
| | 100·0 | 100·0 | 100·0 |

The enormous preponderance of diseases over sickness from injuries, and the great excess of zymotic diseases, "nearly all of which are, in a greater or less degree, preventible by proper precautions," are commented upon by the Commission.

Diseases of malarial type had given rise to much anxiety, but a slight declension was observable. The use of quinine among the troops exposed to malarious influence was recommended by the Commission, and in several instances the adoption of the recommendation had been followed by a marked decrease in the sick list. Typhus was on the increase in the camps. "Its appearance was traceable to the natural disposition of soldiers to shut themselves up in their tents or huts as much and as closely as possible in cold weather. In many camps they have already been allowed to commence a system of suicide, by excavating the ground within their lodgings, and throwing up banks of earth against their walls or curtains. . . . . An extensive outbreak of typhus (adds the Report) would be exceedingly demoralising as well as destructive, and it would be better that double or triple the usual allowance of blankets and flannel shirts should be distributed to the men in camps, even if the issue should be left behind or thrown away at the first movement, than they should be indulged in their disposition to burrow or seal themselves in their lodgings." Measles and smallpox were common, the latter so much so as to justify some uneasiness. The Commission had up to the time of the Report provided for the vaccination of more than 20,000 men.

The Report touches briefly upon the defects in existing and the want of additional military hospitals, and shows that technical and formal obstacles had proved to be almost as serious a draw-back upon the welfare of the sick, as with us at the commencement of the Crimean war. The state of the medical branch of the regular service is discussed, but this we pass over; and the importance of instituting a preliminary examination of the candidates for medical service with the volunteers is insisted upon. In distributing the prodigious quantity of stores entrusted to them for the use of the volunteers, the Commission was cautious that no officer should unnecessarily be relieved from an existing responsibility, to secure for all dependent on him all the supplies which it was his right and duty to demand directly from the Government. Thirty-four thousand four hundred and eighty-one articles of hospital clothing had been distributed from the Washington depôt of the Commission alone, in November, besides a large bulk of unclassified articles. Vast quantities of stores had also been distributed from other depôts.

The prime source of confusion and difficulty in effecting a thorough organization of the volunteer army, was the attempt which had been made suddenly to stretch a system designed to supply the wants of a well-organized army of less than 20,000 men, under thoroughly-trained officers, and to make it sufficient for the wants of 600,000 civilians rushing together in arms, all at once, with no officers acquainted with the forms of administrative duty for an army, but only leading men from among themselves, and of their own selection, to take the duty of officers under that system.

The results of this want of foresight in the heads of departments, as manifested among the troops in camp and in the more immediate vicinity of their supplies, we have seen. Of the results when the forces were removed to a greater, although still not great, distance from those supplies, and brought into action with the enemy, the report of the Commission on the fight at Bull's Run, affords a remarkable illustration. The Commission says:—

"It is manifest that our army, previous to and at the time of the engagement, was suffering from want of sufficient, regularly-provided, and suitable food, from thirst, from want (in certain cases) of refreshing sleep, and from the exhausting effects of a long, hot, and rapid march, the more exhausting because of the diminution of vital force of the troops due to the causes above enumerated. They entered the field of battle with no pretence of any but the most elementary and imperfect military organization, and in respect of discipline little better than a mob, which does not know its leaders. The majority of the officers had, three months before, known nothing more of their duties than the privates whom they should have been able to lead, instruct, and protect. Nor had they, in many cases, in the meantime been gaining materially, for they had been generally permitted, and many had been disposed, to spend much time away from their men, in indolence or frivolous amusement, or dissipation."

The latest paper we have received, published by the Sanitary Commission, is dated, as we have already stated, in January of the present year. The Commission would appear to have found it requisite very greatly to widen its objects, as this paper contains the report of a meeting of the members of the Commission and Associates, resident in New York, for the purpose of discussing a Bill, prepared by the Commission, for the reorganization of the Army Medical Department. *Section* 1 of this Bill provided that there should be (*a*) a Director-General, who should perform the duties now assigned to the Surgeon-General, and such other duties as may be required by law and regulation; (*b*) a Sanitary Inspector-General, who should have the general supervision of all that relates to the sanitary condition of the army; and (*c*) eight Sanitary Inspectors. (*e*) The number of surgeons of the first class was not to exceed forty, of the second class fifty, and of assistant-surgeons one hundred. There were to be also certain medical cadets, not exceeding one hundred in number, and not less than eighteen or more than twenty-three years of age. These cadets were to be admissible to the service after an examination on medical and sanitary science by a board of medical officers, and after three years' continuous medical service they were to be eligible to be examined for promotion. They were to have the rank, pay, and emoluments of the highest grade of non-commissioned officers in the army. (*d*) There were to be as many hospital stewards as the exigencies of the service required. *Section* 2 provided for the election of the Director-General, Sanitary Inspector-General, and Sanitary Inspectors. *Section* 3 provided for promotion by seniority up to the grade of first-class surgeon; but the higher grades were to be filled by election from the whole corps according to peculiar competency. *Section* 4 provided for the formation of the different grades of surgeons from the existing medical staff. *Section* 5 repealed certain regulations as to the rations of surgeons. *Section* 6 provided for the compulsory retirement of medical officers who had attained the age of sixty-five; and *Section* 7 repealed all Acts and parts of Acts inconsistent with these provisions.

The highest grade of assimilated rank attained by the medical officer in the United States service, it would appear, is a colonelcy; but this Bill proposed to give the Director-General the rank, pay, and emoluments of a Brigadier-General; the Sanitary Inspector-General of a Colonel of Cavalry; the Sanitary Inspectors of a Lieutenant-Colonel of Cavalry; the first-class Surgeons of a

Major of Cavalry; the second-class of a Captain of Cavalry; and the Assistant-surgeon of a First Lieutenant of Cavalry.

We add no comments to the foregoing details. We have kept as closely as possible to the text of our authority, and limited ourselves as much as practicable to the barest recital of the facts open to us. In this manner we have thought that we should best indicate the nature and origin of the unique example of sanitary organization presented by the United States Sanitary Commission; in this manner also we believe that we shall best teach the lesson which, if we err not, ought be learned in this country from the operations of that Commission.

We cannot, however, close this article without referring to the rare activity and devotion displayed by the women of the United States in the welfare of the soldiers. What that activity has been is fittest told in the words of the Commission, contained in the appendix to the Report to which we have so frequently alluded:—

"It is hardly just to let this report go forth to the public without a more distinct reference to the deep and earnest, resolute and abiding spirit of patriotism in the women of the country of which the Commission daily receives more tangible evidence than can be conveyed in words. From a backwoods neighbourhood, for instance, comes a box containing contributions of bed clothing and wearing apparel from sixty women and children, the invoice running thus:—'*One pair of stockings from the widow Barber; one quilt, two bottles currant wine, one cheese, Mrs. Barber; two pillow cases and one pair of stockings, Jane Barber; one pair stockings and one handkerchief, Lucy Barber; one pair mittens and Robinson Crusoe, Jedediah Barber;*' and then follow the list of contributions of another family. A few devout words only are commonly added to such a list, but they imply that the donors are ready to give all they possess if it shall be needed to maintain the inheritance of our fathers. Blankets worn in the Revolution, and others taken in the last war with England, heirloom linen, with great-grandmother's hand-marks, and many family treasures, are sent as free-will offerings, with simple prayers that they may contribute to the comfort of some defender of liberty. To the same end, the first ladies of the land, if any are entitled to that appellation, have without cessation, during all the hot summer, been engaged daily in dry, hard, plodding work, sorting, marking, packing goods, and carrying on extended and tedious accounts and correspondence with the precision, accuracy, and regularity of trained merchants. In all there is little character of romantic enthusiasm, but much, and, as the months pass, more and more of deep-seated, abiding, self-sacrificing resolution."

---

NOTE.—The news from Washington, received after this article had gone to press, shows that the worst forebodings of the Commission have been realized, and their hopes and exertions in a great measure rendered vain.

